# Resection of giant malignant solitary fibrous pleural tumor after interventional embolization: a case report and literature review

**DOI:** 10.1186/s13019-022-01881-z

**Published:** 2022-05-31

**Authors:** Kelin Yao, Lvcong Zhu, Liang Wang, Ruiming Xia, Jianfeng Yang, Wenbin Hu, Zhongqiang Yu

**Affiliations:** 1 Department of Radiology, Affiliated Hospital of Shaoxing University (The Shaoxing Municipal Hospital), Shaoxing, 312000 Zhejiang Province China; 2 Medical College of Shaoxing University, Shaoxing, 312000 Zhejiang Province China; 3 Department of Radiology, Shaoxing People’s Hospital, Shaoxing, 312000 Zhejiang Province China; 4 Department of Cardiothoracic Surgery, Affiliated Hospital of Shaoxing University (The Shaoxing Municipal Hospital), Shaoxing, 312000 Zhejiang Province China

**Keywords:** Solitary fibrous tumor of the pleura (SFTP), Pleura, Tumor, Computed tomography

## Abstract

**Background:**

Solitary fibrous tumor of the pleura (SFTP) is a rare mesenchymal tumor that arises at various sites and typically originates from the pleura. Most patients with SFTPs are asymptomatic, unless the tumor is large. Approximately 20% of SFTP cases are malignant. There are few reports on imaging diagnoses and interventional treatments of SFTP. Here, we report a case of a giant SFTP that exhibited malignant behavior and underwent successful resection after embolization of the main supply artery of the tumor.

**Case presentation:**

We report a clinical case of a giant SFTP in a 66-year-old Chinese female patient complaining of chest tightness and cough for more than 2 months. Ten years ago, the patient had undergone a chest CT scan at a local hospital for cough. Computed tomography (CT) had revealed a mass in the right thoracic region, which was misdiagnosed as a pulmonary abscess by CT-guided biopsy. Therefore, the patient did not receive appropriate/complete treatment at that time. She was hospitalized again, because CT showed significant enlargement of the right thoracic mass, which caused her obvious symptoms of discomfort. The pathological results of CT-guided biopsy at our hospital confirmed SFTP. Considering the large size of the tumor and the rich blood supply, some of the main blood vessels were treated with embolization before surgical resection. A large tumor, about 23 cm × 16 cm × 15 cm in size, was then successfully removed by thoracic surgery. The diagnosis of malignant SFTP was confirmed by surgical pathology and immunohistochemistry.

**Conclusion:**

Imaging findings of SFTPs are not characteristic, especially when a tumor is large, the diagnosis is difficult, and the final diagnosis still depends on histological and immunohistochemical examinations. The two-stage surgical treatment described here, which involves first embolization of the main supplying artery of the large tumor and then complete surgical resection, is effective and safe for SFTPs. Whether needle biopsy or vascular embolization is performed, intervention plays a crucial role in the diagnosis and treatment of patients with SFTPs.

## Background

Solitary fibrous tumor of the pleura (SFTP) is a rare mesenchymal tumor that arises at various sites and typically originates from the pleura. Most patients with SFTPs are asymptomatic, unless the tumor is large. Approximately 20% of SFTP cases are malignant. The imaging manifestations of giant SFTPs are complex, varied, and lack characteristics. The imaging diagnosis and surgical resection of SFTPs are challenging. There are few reports on imaging diagnoses and interventional treatments of SFTPs. Here, we report a rare case of a giant SFTP that exhibited malignant behavior and underwent successful resection after embolization of the main supply artery of the tumor.

## Case presentation

**Fig. 1:**
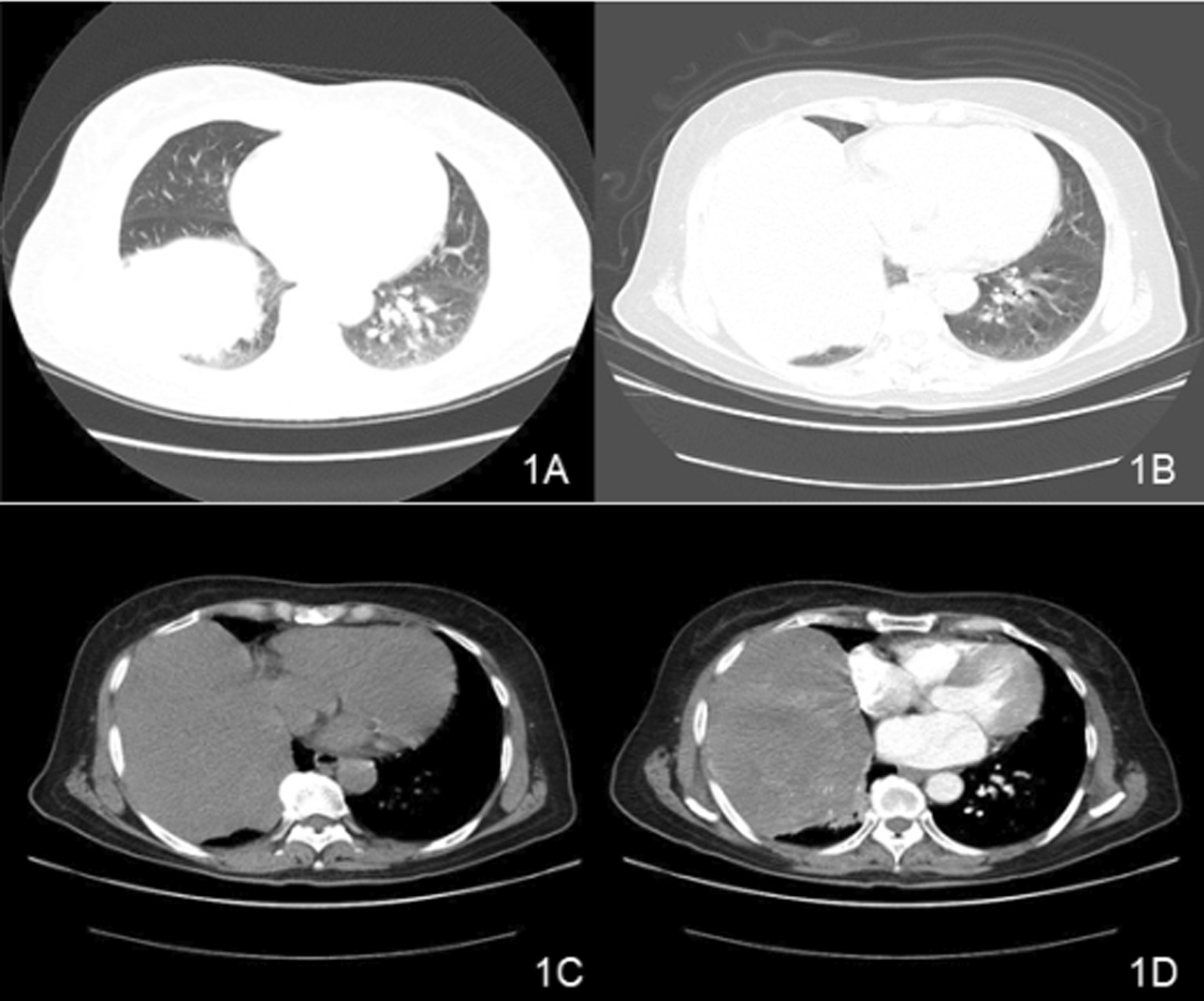
**A** The CT film from 2007. **B** The CT film from 2017 showing the axial lung window; the mass was significantly larger than before. **C** and **D** Large soft tissue mass at the bottom of the right chest, approximately20cm × 13 cm × 12 cm in size, with a clear border and significant enhancement at the axial mediastinal window

**Fig. 2:**
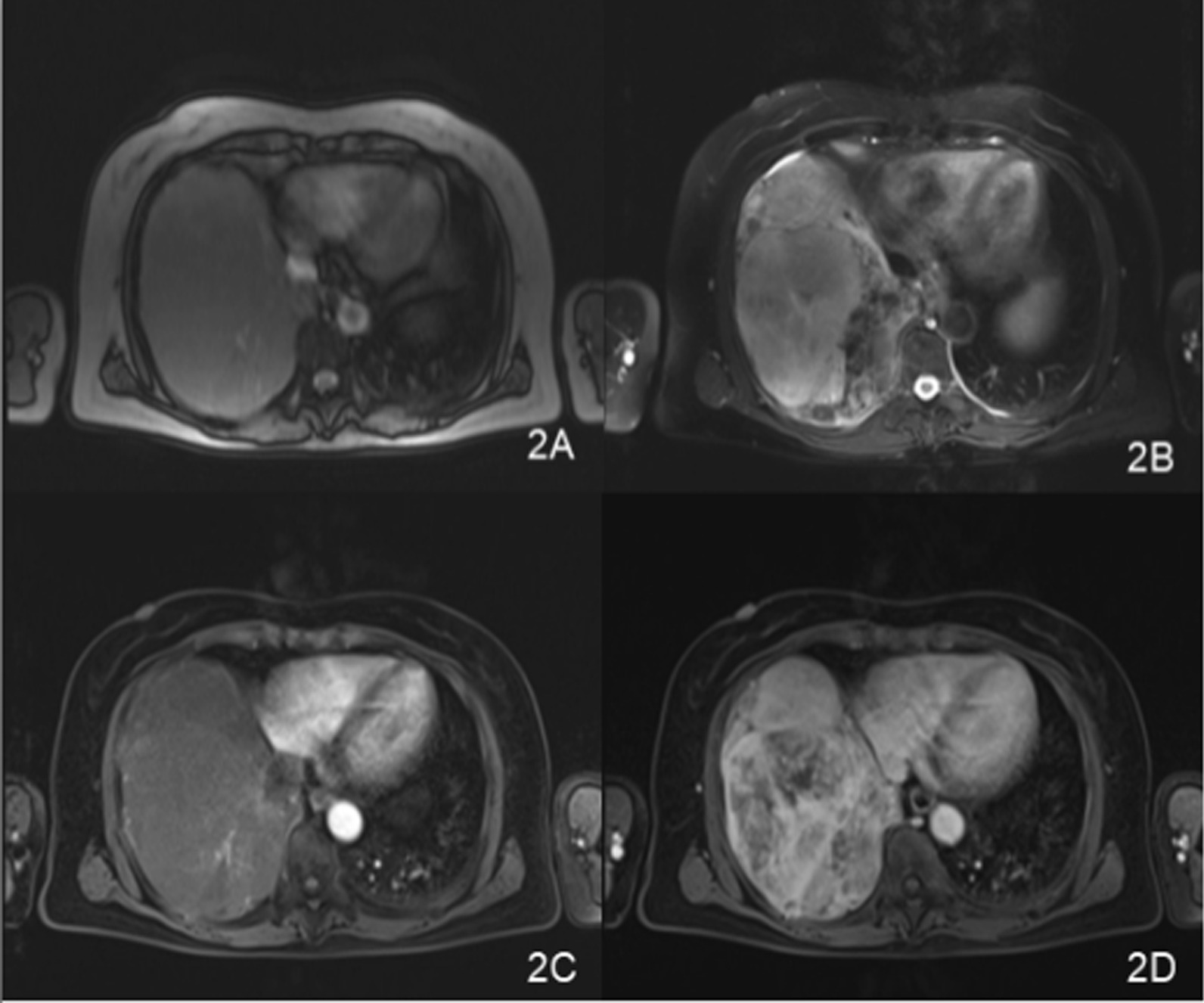
Chest MRI examination showing **A** low signal intensity on T1WI, **B** slightly higher signal intensity on T2WI, and low signal intensity on the internal strip. Contrast-enhanced MR demonstrates obvious heterogeneous enhancement of the lesion, and the necrotic area is not enhanced (**C**, **D**)

**Fig. 3:**
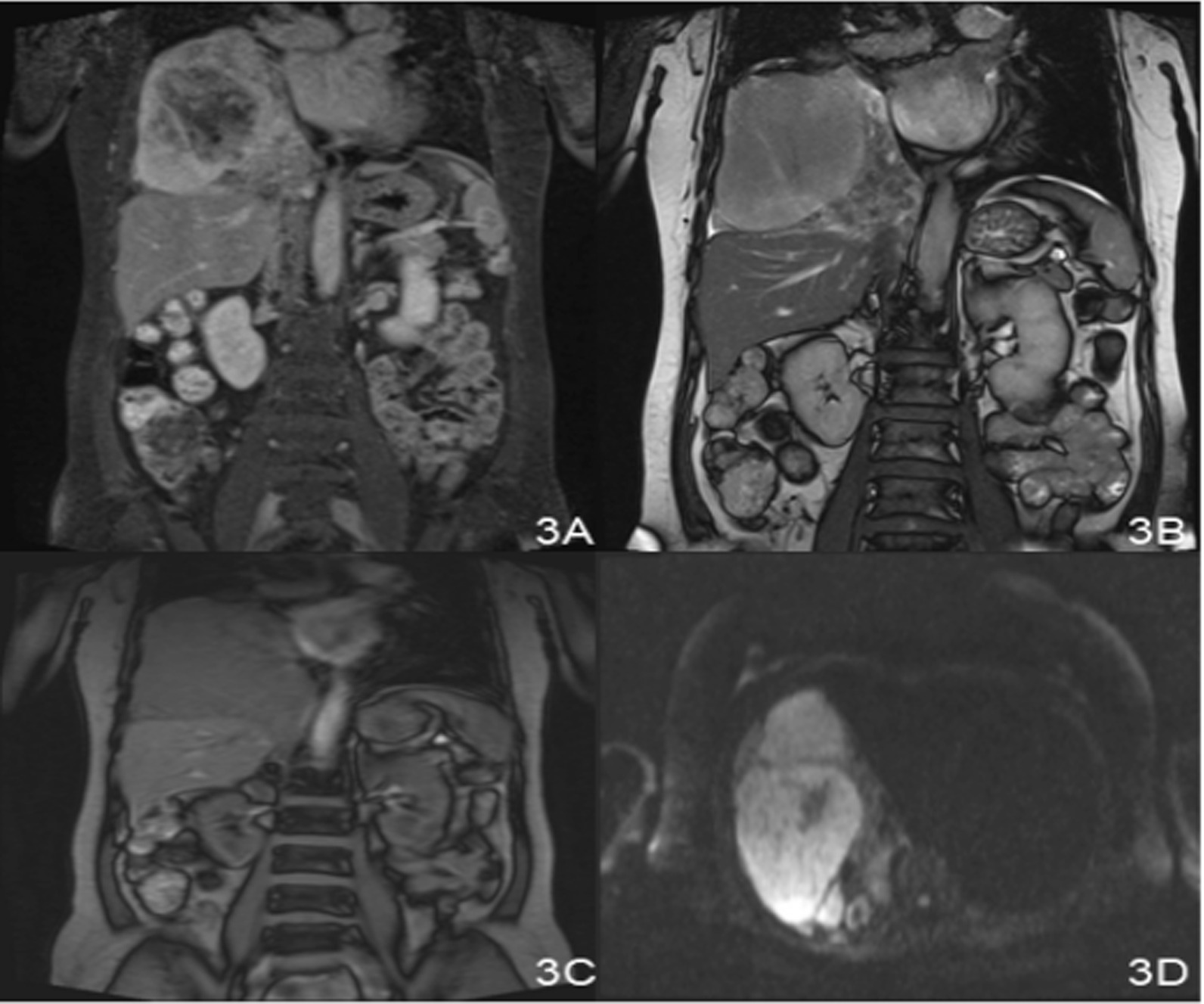
**A**, **B**, **C** Coronal view of chest MRI examination showing the maximum diameter of the mass located in the chest cavity; the right lung lobe is compressed, and the mass has a distinct border with the underlying liver tissue.** D** The mass showing a significant high signal on diffusion weighted imaging (DWI)

A 66-year-old Chinese woman presented with repeated chest tightness and cough for more than 2 months. Computed tomography (CT) examination of her chest revealed a large mass in the right thorax. Ten years ago, the patient had undergone a chest CT scan for a cough, and a mass in the right thorax had been found. CT-guided biopsy of the lesion was performed and it was misdiagnosed as a pulmonary abscess at a local hospital. The patient did not receive complete treatment at the time. She was hospitalized in July 2017 because CT showed that the mass in her right thorax was larger than before, causing obvious discomfort. The patient had no history of occupational exposure to silica, beryllium, or asbestos. Laboratory examination results showed no specificity. Chest CT revealed a significant soft tissue mass at the bottom of the right lung, approximately 20 cm × 13 cm × 12 cm in size, with a clear border. In addition, the mass was significantly larger than before, and contained multiple surrounding nodules (Fig. 1). Chest magnetic resonance imaging (MRI) revealed that the mass had low signal intensity on T1WI, slightly high signal intensity on T2WI, and low signal intensity on the internal strip. Contrast-enhanced CT and MR demonstrated noticeable heterogeneous enhancement of the lesion, but the necrotic area was not enhanced. The coronal view shows that the maximum diameter of the mass was located in the chest cavity, the lung tissue was compressed, and the mass was clearly separated from the lower liver tissue (Fig. 2 and 3). There was no evidence of chest wall or mediastinal involvement and no significant mediastinal displacement.

The imaging appearance of the large chest mass was insufficient to determine the origin and nature of the tumor; therefore, a biopsy was performed. CT-guided biopsy along with immunohistochemical detection revealed that the tumor consisting of spindle cells with local degeneration and necrosis was an SFTP.

**Fig. 4:**
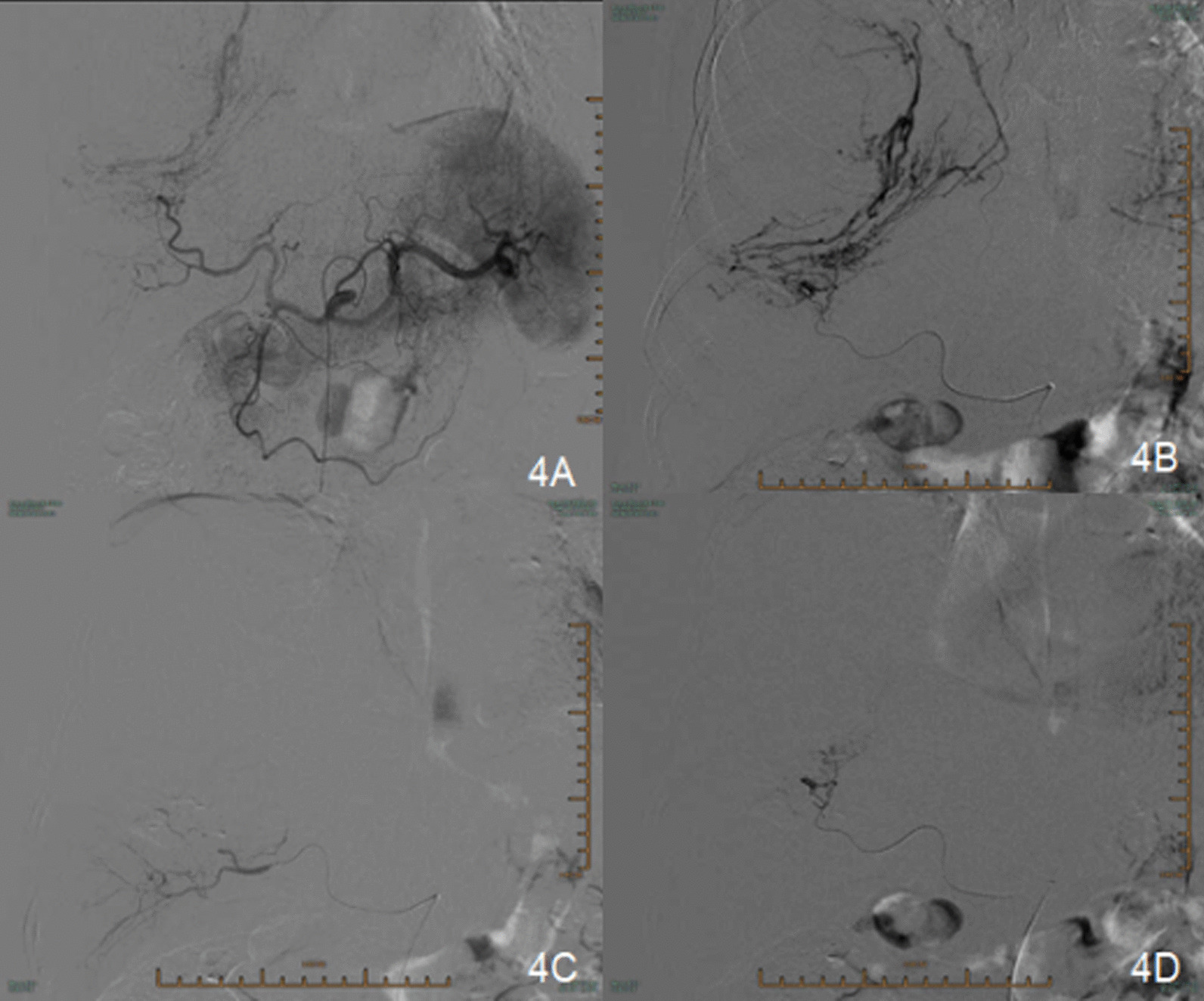
**A**, **B** Angiography confirming that the hepatic artery is involved in the blood supply to the tumor and communicates with the right phrenic artery. **C**, **D** Post-embolization angiography showing that the staining of blood vessels in this part of the tumor has almost disappeared

**Fig. 5:**
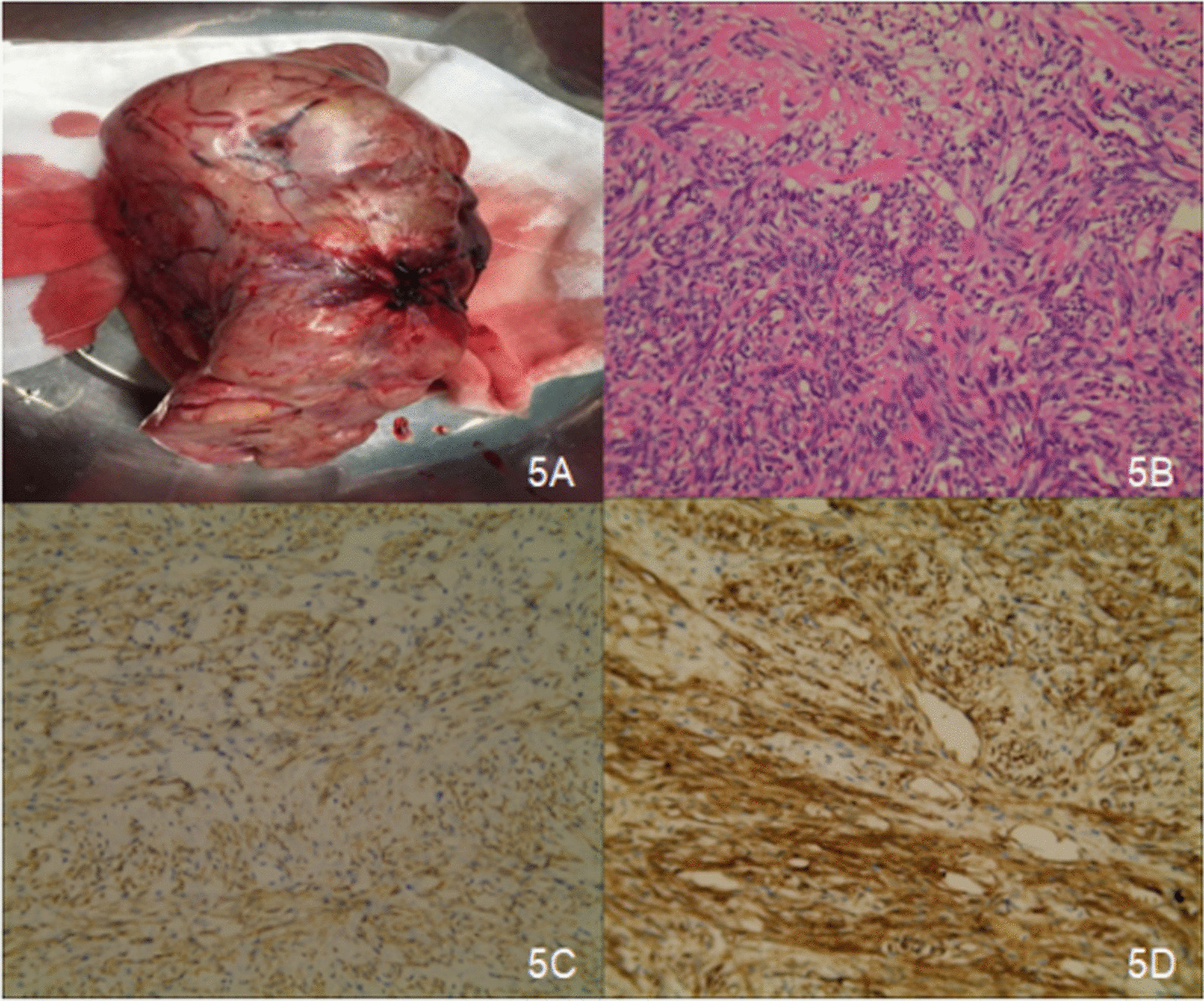
**A**: Surgical excision of the specimen. **B** HE staining at 20 times magnification; tumor cells are abundant, proliferative, and heterotypic (nuclear < 4/10 HPF). **C**, **D** Immunohistochemistry showing positive expression of Bcl-2 and CD34 at 20 times magnification

Because of its large size and the abundant blood supply to the tumor, the patient was referred to our interventional therapy department for preoperative embolization of major blood vessels. After angiographic confirmation, the hepatic artery and the right phrenic artery branches involved in the blood supply to the tumor were embolized (Fig. 4). Right posterolateral thoracotomy through the sixth intercostal space was performed to resect the tumor. Upon entering the pleura, we found that the encapsulated circumscribed massive tumor blocked the view. We chose the ninth intercostal incision space for thoracoscopy and found that the pedicle of the tumor was located at the top of the diaphragm and in the right inferior hemithorax, which was closely related to the lower lobe of the right lung. Finally, the large tumor, approximately 23 cm × 16 cm × 15 cm in size, was completely removed by thoracic surgery. The tumor weighed 2250 g and appeared smooth, surfaced, and well circumscribed on macroscopic examination (Fig. 5). In addition, there were two soft and smooth nodules on the parietal pleura near the thoracic spine. Histologically, the tumor cells were abundant, proliferative, and heterotypic (nuclear < 4/10 HPF). In addition, tumor cell degeneration, necrosis, and an invasive growth pattern were observed on the edge of the tumor. Immunohistochemical evaluation revealed positive staining for CD34, vimentin, and Bcl2, whereas pan-cytokeratin (PCK), smooth muscle actin (SMA), S-100, CD163, CD99, CD68, desmin, calretinin (CR), and human melanoma black-45 (HMB-45) were negative. The tumor was pathologically diagnosed as an SFTP. The right lung expanded completely, and pulmonary function recovered to the normal level after removal of the giant SFTP. The patient had no postoperative complications, and no recurrence occurred during the follow-up period.

## Discussion

Solitary fibrous tumors (SFT) are rare spindle cell tumors originating from dendritic stromal cells that express the CD34 antigen. Both the SFT and hemangiopericytoma (HPC) showed 12q13 inversion and NAB-2 and STAT-6 gene fusion. The two were combined into “SFT/HPC” in the 2016 WHO central nervous system tumor classification [1, 2]. SFTP is a rare neoplasm that accounts for less than 5% of primary pleural tumors. It is derived from the submesothelial mesenchymal layer and usually appears to arise from the visceral pleura, and rarely from the parietal pleura. While benign SFTPs are often small pedunculated tumors, most malignant SFTPs reach > 10 cm in diameter [2, 3]. However, when the tumor diameter is greater than 15 cm or when the tumor occupies > 40% of the hemithorax, it can be defined as a giant SFTP [4]. In the case we describe here, the tumor had been growing for more than 10 years, consistent with the benign tumor growth pattern, until it was finally surgically removed. The maximum diameter of the tumor, which could be regarded as a giant SFTP, was 23 cm. Almost 80% of SFTPs are benign, and more than 50% are asymptomatic. It is generally believed that whether SFTPs cause clinical symptoms may be related to tumor size, and most patients with SFTPs are asymptomatic when the tumor is small. When the SFTP tumor is large enough to cause compression of adjacent structures and lung tissue, patients may experience chest pain, chest tightness, cough, dyspnea, and other symptoms [5, 6]. It is known that some SFTP patients may develop so-called "paraneoplastic syndrome," including refractory hypoglycemia, digital clubbing, and pulmonary hypertrophic osteoarthropathy [7]. In our case, the patient experienced repeated chest tightness and cough for more than 2 months, which is a typical clinical manifestation of SFTPs.

Thoracic CT is the standard radiological modality used to investigate patients with SFTPs. It is a useful diagnostic method that can clearly identify the location and size of the lesion and help surgeons assess the possibility of resecting SFTP. These tumors are usually large, well-defined, lobulated, solid, and vascular masses, often with prominent feeding vessels. The enhancement pattern can vary depending on the cellularity, vascularity, and density of collagenous or fibrous stroma. Central hypoenhancing or non-enhancing areas may be seen in the tumor, which represent necrosis or cystic changes. It often reveals the proliferation of fibrous tissues as well as tumor and adjacent tissue details. Giant SFTPs are usually more likely to cause myxoid or cystic degeneration, hemorrhage, or necrosis. Thus, patchy inhomogeneous enhancement is common in large SFTPs with enhanced scanning. This imaging characteristic is often called “map sign”. The larger the tumor, the more inhomogeneous is the enhancement. Calcification is rare and can be observed in large, benign and malignant tumors [8, 9]. Tumors can also demonstrate remarkable heterogeneity, with variable degrees of enhancement, necrosis, or hemorrhage, and these appearances do not accurately differentiate between benign and malignant lesions. SFTPs > 10 cm in diameter are usually more likely to be malignant [9]. It is difficult to determine the origin of the SFTP tumor when the SFTP tumor is giant and occupies the chest cavity, and MRI multiplane scans may be helpful for localization. MRI can also provide more information for distinguishing between benign and malignant lesions, with heterogeneous signal intensity and contrast uptake found to correlate with malignancy. If mediastinal invasion is suspected, MRI can be useful for surgical planning [10]. In addition, some investigators have suggested that CT-guided aspiration biopsy is not advisable as a reliable tool because of its low diagnostic sensitivity [11]. In our case, the imaging features of the SFTP were consistent with previous literature reports, and there was no significant specificity. However, the patient had undergone CT-guided biopsy of the lesion 10 years ago at a local hospital, and it is possible that the necrotic tissue, which was misdiagnosed as a pulmonary abscess, was obtained by puncture. Such false-negative puncture results are what we must pay attention to.

SFTPs are primary tumors arising from CD34-positive dendritic mesenchymal cells and account for < 5% of all pleural tumors. Immunohistochemically, the SFTP was positive for vimentin, CD34, CD99, and Bcl2 [12]. Thus, tumors tend to grow into huge masses before local compression symptoms develop, especially in patients without routine physical examinations.

## Conclusion

Here, we describe a rare giant SFTP that was successfully treated with surgical resection. In the current case, thoracic CT was essential as a diagnostic imaging method that demonstrated the characteristic patterns of the tumor. Nevertheless, the confirmed diagnosis and differential diagnosis still depend on subsequent histological and immunohistochemical examinations. The first step is to embolize the main blood supply artery and the second step is to remove it surgically. This two-stage surgical treatment approach is highly effective and safe for SFTP [13, 14]. Interventions play a vital role in the diagnosis and treatment of such patients, and long-term follow-ups after surgery are necessary for the early detection of tumor recurrence.

## Data Availability

The datasets used and/or analyzed during the current study are available from the corresponding author upon reasonable request. YKL and YJF will make the data available to the readers.

## Abbreviations

SFTP: Solitary fibrous tumor of the pleura
CT: Computed tomography
MRI: Magnetic resonance imaging
PCK: Pan-cytokeratin
SMA: Smooth muscle actin
CR: Calretinin
HMB-45: Human melanoma black-45
DWI: Diffusion-weighted imaging
